# Integrated care of patients with atrial fibrillation: the 2016 ESC atrial fibrillation guidelines

**DOI:** 10.1136/heartjnl-2016-310843

**Published:** 2017-01-11

**Authors:** Paulus Kirchhof

**Affiliations:** 1 Institute of Cardiovascular Sciences, University of Birmingham, Birmingham, UK; 2 Sandwell and West Birmingham Hospitals NHS Trust and University Hospitals Birmingham Foundation NHS trust, Birmingham, UK; 3 Department of Cardiovascular Medicine, University Hospital Muenster, Germany; 4 Atrial Fibrillation NETwork, Muenster, Germany

**Keywords:** Stroke

Atrial fibrillation (AF) is one of the evolving epidemics in cardiovascular medicine. AF is projected to develop in 25% of currently 40-year-old adults,^1^ ^2^ provokes many, often severe strokes, is associated with increased mortality and often leads to heart failure or sudden death even in well-anticoagulated patients.^3^ ^4^ The last few years have seen the development of new approaches to detect and treat patients with AF, from ECG screening to surgical procedures to prevent recurrent AF. While these improvements provide tools to improve outcomes and quality of life in affected patients, they often need multidisciplinary input into patient management. It therefore seems timely that the European Society of Cardiology (ESC) has issued new AF guidelines in August 2016.^5^

## Good management of patients with AF requires a team effort

Reflecting the need for multidisciplinary input, the 2016 ESC AF guidelines task force consisted of cardiologists with different degrees of sub-specialisation, cardiac surgeons, a stroke neurologist and a specialist nurse, nominated by the ESC and its constituent bodies including the European Heart Rhythm Association, the European Association of Cardio-Thoracic Surgeons and the European Stroke Organization. All recommendations were discussed and voted upon in a predefined process, accepting only recommendations supported by at least 75% of the Task Force members after a structured consultation. Thirty-three general reviewers reviewed the entire guideline in three iterations, while 49 additional reviewers nominated by the ESC National Societies focusing on the class I and III recommendations provided further valuable comments.^5^

## Screening for AF

ECG screening can help detect asymptomatic AF, allowing timely initiation of therapy, especially oral anticoagulation. This has the potential to prevent complications of AF, especially ischaemic strokes. The ESC guidelines recommend opportunistic ECG screening whenever a person aged 65 years or older is seen by a healthcare professional.^6^ In addition, and beyond prior guidelines, systematic ECG monitoring for at least 72 hours is recommended for patients with stroke or transient ischaemic attack (TIA),^7^ ^8^ with a suggestion that even longer ECG monitoring should be considered, especially when a cardioembolic origin of the stroke is suspected.^9^ ^10^ More data are needed to inform the best approach to systematic screening of high-risk population using patient-operated or ‘smart’ devices, with promising initial results in populations aged 75 and above.^11^

## Integrated AF care aligned with patient needs

Once AF is diagnosed, patients should be offered treatment in five domains:

1. acute haemodynamic stabilisation in haemodynamically unstable patients (eg, by cardioversion, rate control, circulatory support, etc);
2. detection and treatment of concomitant cardiovascular conditions;
3. stroke risk assessment and oral anticoagulation in patients with AF at risk for stroke;
4. rate control therapy in patients with elevated ventricular rates during AF;
5. rhythm control therapy in patients who remain symptomatic on rate control therapy.

**Figure 1:**
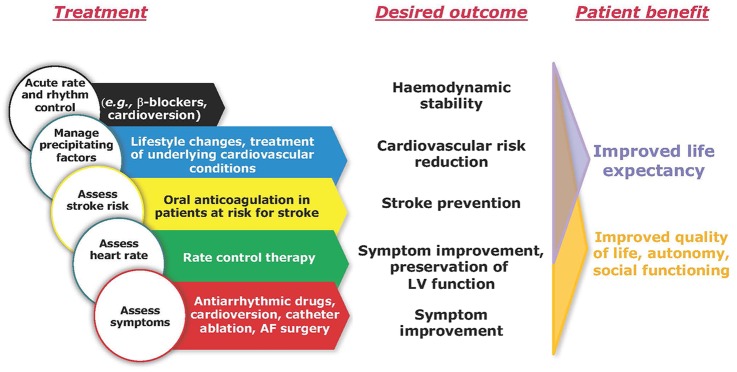
The five domains of integrated atrial fibrillation (AF) care. All patients with AF should receive care directed at these five domains. LV, left ventricular. Reproduced with permission from Ref. 5.

Optimal management of patients with AF requires input from different medical specialties and professions. Integrated care models should be considered for all patients with AF, supporting shared decision-making and empowering patients to own their care, providing support where needed and giving access to all specialist treatment options when appropriate (figure 1).^12^ Such care models will require close cooperation between AF specialists, stroke physicians, AF surgeons, general cardiologists and general practitioners. In addition, continued high-quality care may need further support from allied healthcare professionals, patients, and family members.

## Initial assessment of patients with AF

To detect concomitant cardiovascular diseases, an echocardiogram and a careful analysis of the ECG should be performed in addition to a complete history and physical examination (box 1). This information will often allow us to decide on the need for oral anticoagulation. It will also give a first guide on the need to adapt/initiate rate control therapy, and provide a first idea whether rhythm control therapy is useful.

Box 1 **Clinical information informing atrial fibrillation (AF) management**

▸ Age

▸ Living conditions, personal autonomy (or frailty) and ability to care for oneself

▸ Blood pressure, history of hypertension and antihypertensive therapy

▸ Diagnosed diabetes or current therapy with antidiabetic substances

▸ Prior stroke or transient ischaemic attack

▸ Breathlessness upon exertion, New York Heart Association class (eg, walking distance without breathlessness, ability to climb more than three flights of stairs without a pause), history of heart failure, left ventricular dysfunction, current treatment with heart failure medication

▸ Other vascular disease, eg, carotid artery or peripheral artery disease

▸ Cardiac murmur, history of valvular heart disease or signs of valvular heart disease on echocardiogram

▸ Prior cardiovascular operations or interventions

▸ Exertional chest pain, prior myocardial infarction, percutaneous coronary intervention or bypass surgery, Canadian Cardiovascular Society class

▸ Current anticoagulant or antiplatelet therapy, history of anticoagulation therapy including complications or reasons for interruption

▸ Heart rate on ECG and on palpation, current rate control therapy, prior rate control therapy (digitalis preparations, verapamil/diltiazem, β-blockers)

▸ Symptoms when in AF, frequency of episodes, modified European Heart Rhythm Association score

▸ Current antiarrhythmic drug therapy, prior antiarrhythmic drug therapy, cardioversion, catheter ablation or surgical AF procedures

▸ Other relevant findings on ECG or echocardiogram

▸ Other current medications

## Anticoagulation therapy in AF: aiming for safe and effective long-term therapy

Many patients with AF are now adequately initiated on oral anticoagulation therapy based on the CHA_2_DS_2_VASc stroke risk factors that were included in the 2010 ESC AF guidelines.^13^ ^14^ This established system continues to be recommended: patients with two of the CHA_2_DS_2_VASc stroke risk factors congestive heart failure, hypertension, diabetes or vascular disease, as well as survivors of a stroke or TIA and those aged 75 or more, should receive oral anticoagulation. Patients without any of these stroke risk factors are at low stroke risk and do not need antithrombotic therapy. In patients with one of the CHA_2_DS_2_VASc factors, stroke risk varies,^15^ and female sex does not alter stroke risk in these patients (web addenda Table 1 in Ref. 5,).^15^ ^16^ Consequently, anticoagulation should be considered in patients with AF and one additional stroke risk factor of either sex, weighing the likely benefit of anticoagulation and the risk of bleeding. In selected patients, biomarkers may be useful to refine stroke risk.^17^

## Choice of anticoagulant

Oral anticoagulation to prevent strokes in patients with AF can be achieved with vitamin K antagonists or non-vitamin-K-antagonist oral anticoagulants (NOACs). Patients with mechanical heart valves and those with moderate or severe mitral stenosis should be treated with vitamin K antagonists. Considering their superior safety compared with warfarin (10% lower mortality, approximately 50% lower risk of intracranial haemorrhage or haemorrhagic stroke^18^ ^19^), patients who are eligible for therapy with the NOACs apixaban, dabigatran, edoxaban or rivaroxaban (in alphabetical order) should preferentially be treated with a NOAC.

## Ensuring safe and continued oral anticoagulation therapy

While oral anticoagulation is increasingly used in patients with AF at risk of stroke, many patients with AF are taken off anticoagulants in the first year after initiation. Therefore, a large portion of the guidelines is dedicated to the safe continuation of oral anticoagulation therapy in patients with AF. The guidelines provide a list of four modifiable bleeding risk factors (box 2). Minimising these factors is recommended as a simple way to reduce the risk of major bleeding in anticoagulated AF patients. Furthermore, there are recommendations and flow charts to

1. inform the management of patients with a bleed, including the use of reversal agents;
2. suggest the reinitiation of oral anticoagulation after an ischaemic stroke;
3. support the difficult decisions that need to be taken in patients who suffered an intracranial bleed on oral anticoagulation.

Box 2 **Modifiable bleeding risk factors in anticoagulated atrial fibrillation patients**

▸ Hypertension (especially when systolic blood pressure is >160 mm Hg)

▸ Labile International Normalized Ratio (INR) or time in therapeutic range <60% in patients on vitamin K antagonists

▸ Medication predisposing to bleeding, such as antiplatelet drugs and non-steroidal anti-inflammatory drugs

▸ Excess alcohol (≥8 drinks/week)

▸ Reproduced with permission from Ref.^5^

## A simplified approach to rhythm control therapy in patients with symptomatic AF

There are no major changes to the recommendations for rate control therapy. Rhythm control therapy continues to be indicated to improve AF-related symptoms, usually on top of management of concomitant conditions, stroke prevention therapy and rate control therapy. The indications for antiarrhythmic drug therapy have not changed. The new guidelines provide more detailed information about the ECG changes that are warning signs for proarrhythmia. Catheter ablation targeting isolation of the pulmonary veins^20–22^ is a valid rhythm control therapy option and should be discussed with patients in need for rhythm control therapy. When AF recurs and further rhythm control therapy is warranted, ‘hybrid therapy’ (eg, combining antiarrhythmic drugs with catheter ablation) will often help: in patients who need further rhythm control therapy despite antiarrhythmic drug therapy, catheter ablation is often effective. Patients who need a third attempt to rhythm control therapy should be discussed with an AF Heart Team including consideration of AF surgery.

The new guidelines suggest a very similar approach to rhythm control in patients with paroxysmal (self-terminating) and persistent AF: the flow charts are not different by AF pattern. This simplification is supported by two observations: the time in sinus rhythm as assessed by an implanted device is not that different between patients who are clinically classified as ‘paroxysmal’ or ‘persistent’ AF.^23^ Furthermore, the available controlled trials suggest that catheter ablation is more effective than antiarrhythmic drugs and cardioversion in patients with persistent AF,^24^ similar to paroxysmal AF.

## AF heart teams

Integrated AF care has the potential to provide continued care in the community while maintaining specialist input for most patients with AF (figure 1). Difficult situations, for example, arising in patients who develop a stroke or an intracerebral bleed on anticoagulation, or in patients who need more intensive rhythm control therapy despite initially successful ablation and antiarrhythmic drug therapy, should be discussed in AF Heart Teams. Such teams have already formed, for example, for decisions regarding left atrial appendage occluders, but there seems to be a need to form more local AF Heart Teams.^25^

## Outlook

The 2016 ESC guidelines for AF summarise the current evidence-based therapy for AF in a practical way. Implementing this evidence-based management successfully, probably using integrated AF care models supported by digital tools such as the AF section in the ESC pocket guidelines app, has the potential to substantially improve outcomes in patients with AF. Even on optimal therapy, premature cardiovascular deaths, hospitalisations for cardiovascular problems and diminished autonomy are common. Hence, there is a clear need for further research into the causes of AF, into better ways to prevent AF, the role of rhythm and rate control therapy for AF-related outcomes, and into improved treatment of patients with AF.
